# Molecular and serological characterization of pathogenic *Leptospira* spp. isolated from symptomatic dogs in a highly endemic area, Brazil

**DOI:** 10.1186/s12917-021-02930-w

**Published:** 2021-06-21

**Authors:** Cassia Moreira Santos, Gabrielle Cristini Del Rigo Santos Dias, Alexya Victória Pinheiro Saldanha, Stephanie Bergmann Esteves, Adriana Cortez, Israel Barbosa Guedes, Marcos Bryan Heinemann, Amane Paldês Gonçales, Bruno Alonso Miotto

**Affiliations:** 1 Universidade Santo Amaro, São Paulo, SP Brazil; 2 Departamento de Medicina Veterinária Preventiva e Saúde Animal, Faculdade de Medicina Veterinária e Zootecnia, Universidade de São Paulo, São Paulo, SP Brazil

**Keywords:** Autumnalis, Dogs, Icterohaemorrhagiae, Isolate, MAT, PCR

## Abstract

**Abstract:**

**Background:**

Leptospirosis is an endemic zoonosis in Brazil, with a great impact on human and animal health. Although dogs are frequently infected by pathogenic *Leptospira*, the current epidemiological understanding of canine leptospirosis is mainly based on serological tests that predict the infecting serogroup/serovar. Thus, the present study aimed at identifying the causative agent for severe cases of canine leptospirosis in a highly endemic area through the isolation and characterization of the isolated strains.

**Results:**

Urine, serum and blood samples were collected from 31 dogs with suspected acute leptospirosis treated at the Veterinary Hospital Service of Santo Amaro University between 2018 and 2019. Acute infection was confirmed in 17 dogs (54.8%) by the associated use of Polymerase Chain Reaction (PCR), Microscopic Agglutination (MAT) and bacteriological culture. Eleven dogs (35.5%) had titers ≥800, with the most frequent serogroups being Autumnalis and Icterohaemorrhagiae (n = 4 each) and Canicola (n = 2). Leptospires were recovered from four dogs, and Multilocus Sequence Analysis (MLSA) revealed infection caused by *L. interrogans*, which were further characterized as serogroups Canicola (n = 1) and Icterohaemorrhagiae (n = 3).

**Conclusion:**

The identity of the isolates and serological pattern of MAT suggest that dogs are highly exposed to the serogroup Icterohaemorrhagiae and Canicola, also indicating possible circulation of serogroups not yet isolated in Brazil, notably serogroup Autumnalis. Our findings also reinforce the usefulness of using multiple diagnostic approaches to confirm acute canine leptospirosis.

## Background

Leptospirosis is a bacterial zoonotic disease of worldwide distribution, potentially lethal and capable of causing epidemic outbreaks in human and domestic animal populations [1, 2]. Dogs can act as an important source of infection for humans and other mammals, as they are able to excrete the pathogen into the environment via urine and other body fluids [3]. Therefore, the identification of infected individuals is essential to support the implementation of preventive measures, with a strong One Health impact.

Canine leptospirosis has been widely reported, and infection in dogs can lead to a wide variety of clinical manifestations, ranging from asymptomatic infections or mild febrile illness to severe multisystem organ failure [4, 5]. Laboratory findings are mostly inconclusive, and the definitive diagnosis is still based on confirmatory tests for the direct or indirect identification of the pathogen [6], such as dark-field microscopy, Polymerase Chain Reaction (PCR), bacterial culture and serum antibody titration.

The Microscopic Agglutination Test (MAT) has been widely used for the serodiagnosis of acute disease in dogs, and it is considered the gold standard to confirm leptospiral infection [6]. However, the impossibility to identify the infecting serovar and its inability to differentiate vaccine-induced titers from natural exposure have restricted its use in a clinical setting [7]. Moreover, the evaluation of a single sample may fail to detect antibodies during the initial phase of the disease, and it is often necessary to also test sera obtained in the convalescent phase to reveal seroconversion, which is often not possible due to the high lethality of the disease.

The PCR technique, on the other hand, has been successfully used to confirm infection at the early stages of the disease, showing to be also a useful tool to identify infecting species by further DNA sequencing of PCR amplicons [8–10]. Yet, PCR may not provide reliable results during the convalescent phase of the disease and its use as a single diagnostic strategy is also limited [4].

Since the exact time of infection at clinical presentation is typically unknown, and as both MAT and PCR diagnostic sensitivity and specificity are highly dependent on the stage of the disease, the association of both tests paired with careful clinical and biochemical/hematological evaluation is highly recommended to increase the chances of a definitive diagnosis and further therapeutic success [11]. Yet, despite the benefits of using multiple diagnostic strategies, only the isolation of leptospires and subsequent characterization of the isolates can provide full identification of the infecting strain [6].

Acute disease in dogs is typically associated with serogroups Icterohaemorrhagiae, Canicola and Grippotyphosa, however accidental infection caused by a broader spectrum of serogroups have been described, such as Pomona, Australis, Autumnalis [12], Hebdomadis, Bratislava, Bataviae, Sejroe and Ballum [6, 8, 12–16]. The establishment of a panel of leptospiral strains circulating among local canine populations remains the only and main strategy to subsidize the development and commercialization of polyvalent vaccines that can effectively provide protection for susceptible canine populations, and the wide variety of infecting serovars so far described evidences the importance and the challenges of producing vaccines containing representative leptospires circulating locally.

Unfortunately, isolation is challenging due to the fastidious growth of the pathogen and the frequent contamination of culture media [6]. Furthermore, culturing is frequently restricted by the early institution of antimicrobial therapy, which is usually implemented immediately after the disease is suspected [11], and the low sensitivity of the technique undermines clinicians to even consider culturing as a diagnostic strategy during the clinical procedure.

Even in face of the importance of characterizing leptospires for further vaccine development, few studies tackling the causative agent of canine leptospirosis have been conducted in Brazil [4, 17–21] and the characterization of leptospires isolated from dogs with clinical suspicion of the disease remains poorly documented.

Dogs may act as important sentinels to investigate serovars circulating in certain regions and surveillance of leptospiral strains causing clinical disease in dogs should be further explored, especially in locations with rural-urban interfaces, where contact with wildlife fauna predisposes infection caused by unusual leptospiral strains.

By using an integrative approach, which included clinical/laboratorial evaluation and serological and molecular methods, the present study aims to provide characterization of the isolated leptospires from dogs with acute leptospirosis treated at the Veterinary Service of the University Santo Amaro (UNISA), a hospital service that provides animal care for low-income communities located at the most endemic region for human leptospirosis in São Paulo city, Brazil.

## Results

Out of the 31 dogs included in the study, 20 (64.5%) had anti-*Leptospira* antibodies detectable by MAT, with titers ranging from 100 to 12,800. Only two dogs were recently vaccinated (19 and 29), but dog 29 had no detectable antibodies. Eleven dogs had acute infection determined by MAT (35.5%), as shown in Table 1. Ten of them had titers ≥800 in a single serum sample, while serodiagnosis of one dog (dog 10) was established by MAT titration of convalescent serum samples, which showed seroconversion against Icterohaemorrhagiae serogroup, shown in Table 2.

**Table 1:** Outcome, culture, PCR, MVLA, MAT results and immunization records found in 31 dogs suspected of acute leptospirosis.

Dog ID	Clinical Outcome	Isolation	Blood PCR	Urine PCR	MVLA (presumptive serogroup)	MVLA (DNA origin)	MAT-confirmed cases	Highest Serovar titer found	Vaccination (<12 months)
1	Died	−	−	NP	NP		−	−	−
2	Died	−	(+)	(+)	−		−	−	−
3	Died	−	−	−	NP		−	−	−
4	Died	(+)	(+)	(+)	ICT	Isolate	−	−	−
5	Died	(+)	(+)	NP	ICT	Isolate	−	−	−
6	Died	(+)	−	(+)	CAN	Isolate	(+)	CAN	−
7	Survived	−	−	−	NP		−	−	−
7^a^		−	−	−	NP		−	−	
8	Died	−	(+)	NP	−		(+)	BUT	−
9	Died	(+)	(+)	(+)	ICT	Isolate	(+)	ICT	−
10	Survived	−	−	(+)	−		−	−	−
10^a^		−	−	−	NP		(+)	ICT	
11	Died	−	−	−	NP		−	−	−
12	Died	−	−	−	NP		(+)	CAN	−
13	Died	−	(+)	NP	ICT	Blood sample	−	−	−
14	Died	−	−	−	NP		(+)	COP	−
15	Died	−	−	NP	NP		−	−	−
16	Died	−	(+)	−	ICT	Blood sample	−	−	−
17	No follow-up	−	−	−	NP		−	−	−
18	No follow-up	−	−	−	NP		−	−	−
19	Survived	−	−	−	NP		(+)	Coagglut.	(+)
19^a^		−	−	−	NP		−	−	
20	No follow-up	−	−	−	NP		−	−	−
21	Survived	−	−	−	NP		−	−	−
21^a^		−	−	−	NP		(+)	COP	−
22	Died	−	−	−	NP		−	−	−
23	Died	−	−	−	NP		(+)	BUT	−
24	No follow-up	−	−	−	NP		−	−	−
25	Died	−	−	(+)	−		(+)	BUT	−
26	Died	−	−	(+)	−		(+)	BUT	−
27	Died	−	−	−	NP		−	−	−
28	Survived	−	−	−	NP		−	−	−
28^a^		−	NP	NP	NP		−	−	
29	Died	−	−	−	−		−	−	(+)
30	Survived	−	−	(+)	ICT	Urine sample	−	−	−
31	Died	−	−	NP	NP		−		
TOTAL		**4**^b^	**7**^b^	**8**^b^			**11**^b^		**2**^b^

*(+)* positive, *−* negative, *NP*, Not performed, *BUT* serovar Butembo, *CAN* serovar Canicola, *COP* serovar Copenhageni, *ICT* serovar Icterohaemorrhagiae, *Coagllut* Coagglutination

^a^Revaluations

^b^The total considers only the amount of positive results per column

**Table 2:** MAT titers and serovars found in 31 dogs suspected of acute leptospirosis

Serogroups, serovars and respective titer found in MAT														
Dog ID	AUS		AUT		BAL	BAT	CAN	CYN	GRI	ICT		POM	PYR	SHE
	AUS	BRA	AUT	BUT	CAS	BAT	CAN	CYN	GRI	COP	ICT	POM	PYR	SHE
**1**	−	−	−	−	−	−	−	−	−	−	−	−	−	−
**2**	−	−	−	−	−	−	−	−	−	200	−	−	−	−
**3**	−	−	−	−	−	−	100	−	−	−	−	−	−	−
**4**	−	−	−	−	−	−	−	−	−	−	−	−	−	−
**5**	−	−	−	−	−	−	−	−	−	−	−	−	−	−
**6**	−	−	−	800	1600	−	3200	−	−	200	200	−	−	200
**7**	−	−	−	−	−	−	−	−	−	−	−	−	−−	−
**7**^a^	−	−	−	−	−	−	−	−	−	−	−	−	−	−
**8**	−	−	−	800	−	−	−	200	−	200	−	−	−	200
**9**	−	−	−	400	−	−	−	−	−	200	800	−	−	200
**10**	−	−	−	−	−	−	−	−	−	−	−	−	−	−
**10**^a^	800	−	800	400	100	−	200	200	−	200	12800	1600	−	6400
**11**	−	−	−	200	−	−	−	−	−	−	400	−	−	400
**12**	−	−	−	−	−	−	800	−	−	−	−	−	400	−
**13**	−	−	−	−	−	−	−	−	−	−	−	−	−	−
**14**	−	−	−	200	−	−	−	−	−	12800	6400	−	−	6400
**15**	−	−	−	−	−	−	−	−	−	100	−	−	−	−
**16**	−	−	−	100	−	−	−	−	−	400	−	−	−	−
**17**	−	−	−	−	−	−	−	−	−	200	200	−	−	200
**18**	−	−	−	−	−	−	−	−	−	100	100	−	−	100
**19**	−	1600	−	1600	−	800	−	−	−	800	200	−	−	800
**19**^a^	−	400	−	−	−	−	−	−	−	200	200	−	−	−
**20**	−	−	−	−	−	−	−	−	−	−	−	−	−	−
**21**	−	200	−	−	−	−	200	−	−	400	200	−	−	200
**21**^a^	−	400	−	−	−	−	200	−	−	800	100	−	−	400
**22**	−	−	−	−	−	−	−	−	−	−	−	−	−	−
**23**	−	400	100	3200	−	−	1600	−	1600	200	400	−	−	−
**24**	−	−	−	−	−	−	−	−	−	100	−	−	−	−
**25**	−	−	−	800	−	−	−	−	−	100	200	−	−	−
**26**	−	−	−	800	−	−	−	−	−	−	−	−	−	−
**27**	−	−	−	−	−	−	−	−	−	−	−	−	−	−
**28**	−	−	−	100	−	−	−	−	−	200	200	−	−	−
**28**^a^	−	−	−	−	−	−	−	−	−	−	100	−	−	−
**29**	−	−	−	−	−	−	−	−	−	−	−	−	−	−
**30**	−	−	−	−	−	−	−	−	−	−	−	−	−	−
**31**	−	−	−	−	−	−	−	−	−	−	−	−	−	−

*AUS* Australis, *BRA* Bratislava, *AUT* Autumnalis, *BUT* Butembo, *BAL* Ballum, *CAS* Castellonis, *BAT* Bataviae, *CAN* Canicola, *CYN* Cynopteri, *GRI* Grippotyphosa, *ICT* Icterohaemorrhagiae, *COP* Copenhageni, *POM* Pomona, *PYR* Pyrogenes, *SEJ* Sejroe, *SHE* Shermani, *−* negative

^a^Revaluations

The serological profile of the MAT-confirmed cases showed that the most frequent serogroups found in the infected dogs were Autumnalis and Icterohaemorrhagiae (n = 4 for each), followed by Canicola (n = 2) (Table 2). One animal presented the highest titers against serogroups Australis and Autumnalis (dog 19), but only titers against Australis persisted after serological reevaluation of the convalescent sample, thus reflecting a possible decrease in titers typically found during the late convalescent phase, or even a paradoxical titration found in the first evaluated sample.

**Fig. 1:**
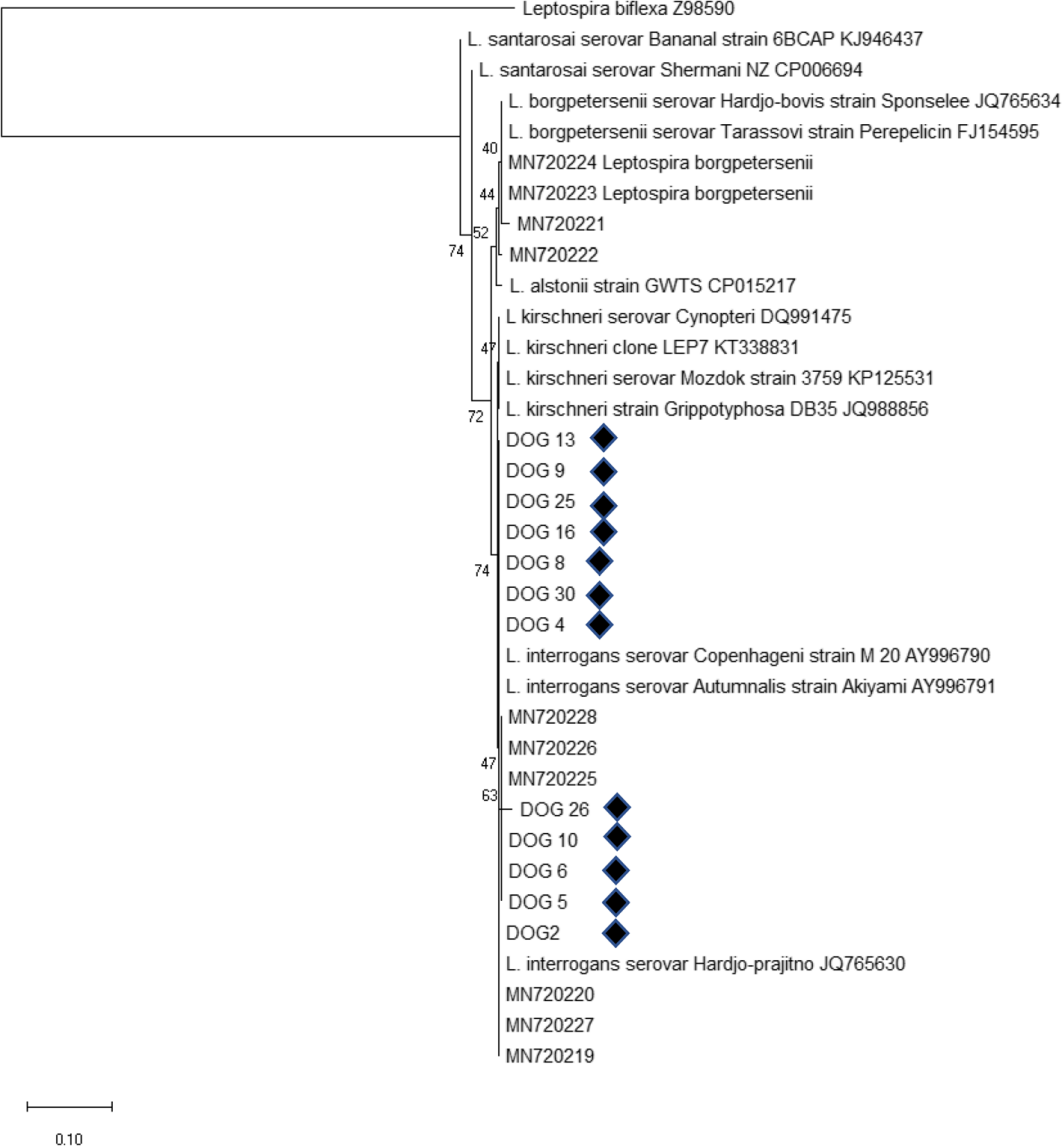
Phylogenetic reconstruction based on partial ribosomal 16S gene sequences of reference strains of *Leptospira* spp. and sequences obtained from dogs with clinical suspicion of acute leptospirosis (black diamonds). The tree was constructed with the Neighbor-Joining method using Tamura-3 parameter model with a bootstrap test of 1000 replicates

*Leptospira* DNA was detected by PCR in 12 dogs (38.7%). Five dogs presented DNA only in urine samples, while four dogs presented DNA exclusively in blood samples, and three dogs yielded positive results in both samples. Twelve PCR-positive dogs had infection confirmed by DNA sequencing, and all sequences presented high similarity (>99%) to *L. interrogans* representatives (AY996798, AY996800). Species identity was further confirmed by the 16S rRNA phylogenetic analysis, which showed the formation of a cluster with reference sequences of *L. interrogans* (Fig. 1). The recovered sequences were submitted to GenBank under accession numbers MW263930 - MW263941.

The comparison between MAT and PCR results showed that five dogs (12, 14, 19, 21 and 23) were diagnosed with MAT, when used as a single diagnostic strategy, and six other dogs (2, 4, 5, 13, 16 and 30) were positive only by PCR, while six animals (6, 8, 9, 10, 25 and 26) were positive in both tests, totaling 17 dogs with confirmed acute infection.

Leptospires could be recovered from four dogs. Three out of four (Strains 117, 118 and 120, recovered from dogs 4, 5 and 9 respectively) showed a specific reaction against serogroup Icterohaemorrhagiae (6400) after serogrouping with polyclonal antibodies, while the strain 119 (recovered from dog 6) presented the highest titration against serogroup Canicola (1600).

The MLVA analysis of the isolates using three discriminatory markers for the VNTR loci 4, 7 and 10 revealed a VNTR 2-1-8 profile in samples taken from dogs 4, 5 and 9, being compatible with the serogroup Icterohaemorrhagiae (serovar Copenhageni, strain Fiocruz L1-130), and a 1-10-3 VNTR profile for the dog sample 6, compatible with the serogroup Canicola pattern (serovar Canicola strain Hond Utrecht IV). The MLVA technique was also applied to positive PCR samples in which the recovery of leptospires was not possible, in an attempt to compare the pattern of clinical samples with those found in the isolates; nevertheless, only samples from dogs 13, 16 and 30 yielded interpretable profiles, which were compatible with the pattern assigned to the serogroup Icterohaemorrhagiae (serovar Copenhageni, strain Fiocruz L1-130).

The clinical outcome from 31 dogs could be determined: six dogs survived after proper treatment. Death or euthanasia occurred in 21 cases, and the overall lethality among cases with confirmed acute infection was 76.5%. The outcome could not be determined in four cases due to the discontinued follow-up care.

## Discussion

Viable leptospires could be recovered from four dogs with confirmed acute leptospiral infection, and further molecular and serological characterization enabled the identification of *L. interrogans* serogroups Icterohaemorrhagiae (strains 4, 5 and 9) and Canicola (strain 6) as causative agents for canine leptospirosis in the present study.

Despite canine acute infection caused by pathogenic *Leptospira* being largely reported in Latin America, the characterization of the infecting strains at a serovar or serogroup level is restricted to a few studies that have successfully isolated the pathogen in culture media [4, 14, 18, 19, 22–27]. In recent years, isolation and proper characterization of the infecting strains in dogs with acute leptospirosis have shown that dogs can be exposed to a wide variety of serogroups [6], depending on the geographical region (e. g. urban or rural settings, tropical or temperate climate), local environmental/behavioral conditions (contact with wildlife fauna, sanitation, restriction of roaming behavior) and implementation of preventive measures against infection (use of vaccines, rodent control policies). Such variety may impose serious challenges to produce effective vaccines containing leptospiral strains circulating locally.

Furthermore, several countries have reported an antigenic shift due to the extensive use of vaccines [8], giving rise to serovars not included in traditional vaccine compositions, highlighting the importance of using dogs as sentinels to access strains circulating locally. In Brazil, the use of multivalent vaccines containing Icterohaemorrhagiae, Canicola, Grippotyphosa and Pomona is predominant, although most dogs included in the study had not been vaccinated at least 12 months prior to clinical presentation. One of the vaccinated dogs (dog 19) had been vaccinated approximately 11 months prior to sample collection. Several reports have demonstrated that MAT titers associated with vaccination only persist for approximately 4 to 6 months [28–30], therefore, is highly unlikely that MAT titers found in dog 19 are related to vaccination.

Our results corroborate previous serological findings showing that Icterohaemorrhagiae is likely the main causative pathogen for acute canine infection in Brazil [4, 19, 31, 32]. These findings are also in agreement with the few studies that have consistently characterized Icterohaemorrhagiae as the main infecting serogroup in symptomatic dogs [4, 19], reinforcing the hypothesis that dogs are highly exposed to environmental contamination promoted by rodents.

Serogroup Icterohaemorrhagiae is also considered the main causative agent for human leptospirosis in Brazil, notably in São Paulo city [33, 34]. The UNISA veterinary hospital is located in the South area of São Paulo, a region with the highest incidence rates for human leptospirosis, with an annual average of 57 reported human cases of leptospirosis between 2017 and 2019 [35]. The area is characterized by having the highest precipitation rates in the city [36], with many humans living in low-income communities with poor sanitation, which can predispose not only humans but also dogs to environmental exposure to pathogenic *Leptospira* spp. Under such conditions, dogs can act as important sentinels [1], and the diagnosis of acute canine infection and further characterization of the infecting *Leptospira* spp. may assist local health authorities to prevent leptospiral transmission by mitigating risk factors shared both by humans and dogs. Moreover, most districts in the area have a rural-urban interface configuration, with intense wildlife fauna interaction, which can lead to the transmission of leptospires maintained by wild reservoirs, reinforcing the importance of implementing local epidemiological surveillance using dogs as sentinels. In fact, a recent serological survey conducted by Diodato et al. [37] has found that Autumnalis is the most frequent serogroup found in asymptomatic semi-domiciled and stray dog populations from the South area of São Paulo. Interestingly, even in face of the successful characterization of Icterohaemorrhagiae and Canicola isolates in the present study, several dogs with acute infection confirmed by MAT presented the highest titers against Autumnalis serogroup.

Acute infection caused by Autumnalis was recently reported in humans and dogs from Japan, thus indicating a possible zoonotic transmission of this particular serogroup. In Latin America, serological surveys conducted in the islands of Barbados, Saint Kitts and Trinidad have also found Autumnalis as the predominant serogroup among dogs, and the isolation of serovar Bim (serogroup Autumnalis) from an asymptomatic carrier dog has also been described [23, 38–41].

In Brazil, Autumnalis was identified as the main infecting serogroup found in stray and asymptomatic dog populations from different parts of the country [42–45], however, confirmation of infection by isolation and molecular/serological characterization hasn’t been described yet. Unfortunately, isolation was not achieved in samples taken from dogs presenting the highest titers against Autumnalis in the present study, and the actual infecting serogroup attributed to those cases could not be determined. The MLVA analysis has also failed to identify serogroup identity in these samples, and despite *L. interrogans* infection was confirmed by 16SrRNA sequencing analysis, molecular typing methods using single-gene sequencing have poor discriminatory power to identify leptospires at a serovar/serogroup level. Therefore, serological evidence of Autumnalis infection should be interpreted with caution, as MAT results might have a low correlation with the actual infecting serogroup due to cross-reaction between serovars [46, 47], especially in acute cases, where a paradoxical reaction is common. Moreover, cross-reactions between serogroup Icterohaemorrhagiae and Autumnalis have been described after natural infection by Icterohaemorrhagiae serogroup, and the use of commercial vaccines containing Icterohaemorrhagiae representatives may also potentially induce cross-reactions between both serogroups [48–50].

In the present study, the association of PCR and MAT tests allowed the diagnosis of leptospirosis in 17 of the 31 dogs included in the study (54.8%), demonstrating that part of these animals would have had a false-negative result if only one of the tests had been performed. Despite the benefits of associating both tests for the diagnosis of acute canine leptospirosis, the PCR was able to diagnose more infected dogs when used as a single strategy, thus indicating the benefits of this method for the definitive diagnosis at the early stages of the disease, as previously reported [4, 51].

The PCR results from 12 positive samples were confirmed by sequencing and phylogenetic analysis, and all sequences clustered with sequences belonging to *L. interrogans* representatives. This approach has allowed the identification of unexpected leptospires infecting dogs in Brazil, such as *L. santarosai*, *L. kirchnneri* and *L. noguchii*, evidencing that this approach could be useful to circumvent the role of dogs in the transmission chain of leptospirosis [4, 21, 52].

The MLVA analysis has confirmed species identity of the isolates, with 100% agreement with MAT using polyclonal antibodies. This molecular technique has been successfully used to identify particular serogroups in clinical samples in which isolation could not be achieved [53]. In the present study, most samples presenting leptospiral DNA confirmed by the 16SrRNA PCR protocol have not yielded MLVA positive results, probably due to the insufficient DNA concentration. Nevertheless, MLVA was able to identify infection by serogroup Icterohaemorrhagiae in two dogs with no MAT titers, and the application of this technique should be incorporated in future studies tackling canine acute leptospiral infection.

Despite the usefulness of molecular methods for the diagnosis of acute leptospirosis, the use of PCR may lead to false-positive results in cases where non-leptospiral diseases mimicking acute leptospirosis are concomitantly present in dogs presenting chronic urinary shedding of leptospires. Moreover, no proper comparison between MAT and PCR results could be performed in consequence of the high lethality of the disease, which hampered the evaluation of MAT convalescent titers in most dogs.

The lethality was strikingly high among the suspected cases studied (67.7%), reinforcing previous studies from our group [4]. These findings highlight the urgent need for implementing new therapeutic strategies in canine leptospirosis, as observed in clinical trials with human subjects [54]. Additionally, the high proportion of dogs with no confirmation of infection reveals that investigation of other causes leading to clinical signs similar to acute leptospirosis should be investigated, and that the current diagnostic strategies should be improved for the diagnosis of the disease.

## Conclusion

Our results have demonstrated that dogs from São Paulo South region are being exposed to serogroup Icterohaemorrhagiae, Canicola and possibly serogroup Autumnalis. Our findings also reinforce the usefulness of using multiple diagnostic approaches to confirm acute canine leptospirosis, in order to mitigate false-negative serological results and false positive results attributed to vaccination. The use of leptospiral culture and further molecular and serological analyses may assist the identification of the infecting leptospires, revealing if serovars included in the vaccines used locally actually correspond to the circulating strains. The extensive use of bivalent vaccines including Icterohaemorrhagiae and Canicola serovars is highly recommended to prevent acute canine leptospirosis and possible zoonotic transmission in the studied region, and future studies should be addressed to confirm the presence of serovar Autumnalis in São Paulo South region.

## Materials and method

### Study area

The veterinary hospital of the Santo Amaro University is located in the southern region of the city of São Paulo (Capela do Socorro sub-prefecture), a region composed of approximately 173,180 households, of which 31,896 have no access to sewage system. The region has 594,930 m^2^ of green area, characterized by an extensive rural-urban interface bathed by two important large river dams [55].

### Experiment design and inclusion criteria

Urine and whole blood samples were collected from 31 dogs with clinical suspicion of leptospirosis treated at the Veterinary Hospital of Santo Amaro University (HOVET-UNISA), between September 2018 and November 2019. Inclusion criteria consisted of dogs presenting blood urea nitrogen and creatinine levels above the reference values (64.2 mg/dL and 1.4 mg/dL, respectively) or jaundice, that also presented two or more clinical manifestations suggestive of acute leptospirosis, including hemorrhagic disorders, fever, vomiting, prostration, hyporexia/anorexia and oliguria/anuria. Vaccination status was also investigated during physical examination, and dogs with a history of polyuria, polydipsia and weight loss one month prior to the clinical presentation were not included to rule out possible chronic kidney disease.

Blood and urine samples were used for bacterial culturing and DNA detection of leptospires by PCR. Serum samples were destined for the microscopic agglutination test (MAT) and serum biochemistry analyses. The samples taken at first medical assistance were collected between clinical/laboratorial suspicion of the disease and the establishment of antimicrobial therapy.

The dogs were prospectively monitored after the first evaluation, and revaluations included serum detection of anti-*Leptospira* antibodies to detect possible seroconversion. Revaluations were carried out up to 14 days after the first evaluation, with no systematized sampling scheme.

### Diagnostic criteria

Dogs that had ≥800 titers detected by MAT, positive PCR and/or bacterial isolation were considered to be in the acute phase of *Leptospira* infection. Dogs that were revaluated and presented seroconversion characterized by a 4-fold increase in antibody titration were also considered actively infected, as well as those that presented no antibodies at the first evaluation but had titres equal to 100 at the second evaluation.

### Collection and storage of samples

Blood samples were collected by venipuncture of the jugular or cephalic veins into EDTA tubes and haematological analyses were performed immediately after collection. Part of the blood samples were drawn into anticoagulant-free tubes to obtain blood sera. Urine samples were collected by cystocentesis to minimize bacterial contamination and increase the chances for culturing leptospires in a selective medium. The blood and urine aliquots destined for bacterial culturing were processed immediately after collection. Urine, blood and serum samples destined to PCR, MAT and serum biochemistry analyses were stored at −20 °C until processing.

### Microscopic agglutination test

The microscopic agglutination test was performed as described by Faine et al. [56]. The serum of the suspected animals was tested against a panel of 20 serogroups including 24 serovars: Australis, Bratislava, Guaricura, Autumnalis, Butembo, Castellonis, Bataviae, Canicola, Whitcombi, Cynopteri, Grippotyphosa, Hebdomadis, Copenhageni, Icterohaemorrhagiae, Javanica, Panama, Pomona, Pyrogenes, Hardjo (Hardopratijno), Hardjo (Hardjobovis), Shermani, Tarassovi, Pomona and Sentot. The cutoff for a positive agglutination reaction was defined as a titer ≥100, however only dogs with ≥800 titers or presenting seroconversion were considered to have acute leptospirosis. Seroconversion was defined as a fourfold (two titre steps) or greater rise in MAT found in paired samples, collected one to two weeks apart. It also included dogs presenting an initial antibody-negative result with a convalescent titer of at least 800 to one or multiple serovars. For each positive sample, the most probable infecting serogroup was the one presenting the highest titers.

### *Leptospira* spp. culturing

For *Leptospira* isolation, 500 μL of urine and whole blood were diluted in sterile Sorensen buffer solution at 1:10 and 1:100, and further seeded into tubes containing Fletcher and EMJH medium (Difco Laboratories, Franklin Lakes, NJ, USA). The tubes were incubated at 28 °C and observed weekly for six weeks to check the superficial growth ring (Dinger’s zone). The presence of leptospires was confirmed by dark-field microscopy visualization.

### DNA extraction and PCR amplification

The urine samples were centrifuged at 13,000×*g* for 15 minutes at 20 °C, and the sediment was resuspended in 300 μL of sterile Tris-EDTA solution after discarding the supernatant. DNA was extracted from 200 μL of blood or resuspended urine sediment using PureLink*®* Genomic DNA Mini Kit (Invitrogen, Thermo Fisher Scientific Inc., Carlsbad, CA, US) following the manufacturer’s recommendations. DNA amplification of pathogenic leptospires was carried out with qualitative assays targeting the 331 bp fragment of the 16S rRNA gene using primers and amplification conditions described by Mérien et al. [57].

The amplified products were separated by electrophoresis on a 1.5% agarose gel stained with SYBR Safe DNA (Invitrogen, Thermo Fisher Scientific Inc., Carlsbad, CA, US) and analysed under ultraviolet transillumination. The amplicons presenting a single band were purified using 2 μL ExoSap-IT (Thermo Fisher Scientific Inc., Carlsbad, CA, EUA) and amplification showing multiple gel bands were purified using the Wizard ® SV gel and PCR Clean-up System kit (Promega Corporation) according to the manufacturer’s specifications.

### Characterization of the isolates and PCR amplicons

#### Serogrouping

The serogroups of the isolates were determined by MAT as previously described [58] using a panel of rabbit anti-sera for 23 polyclonal antisera against *Leptospira* spp., which represented 17 distinct serogroups: Australis, Autumnalis, Ballum, Bataviae, Canicola, Cynopteri, Grippotyphosa, Hebdomadis, Icterohaemorrhagiae, Mini, Panama, Pyrogenes, Pomona, Sarmin, Sejroe, Shermani and Tarassovi. The highest titer for a particular antiserum was used as criteria to identify the presumptive serogroup.

#### DNA Sequencing and phylogenetic analysis

The 16S rRNA amplicons of the positive PCR samples (including samples that leptospires could be also isolated) were sequenced using the *BigDye Terminator Cycle Sequencing kit* (Life Technologies®) for further sequencing analysis. The amplicons were sequenced in an ABI 7500 *Genetic Analyzer* (Life Technologies®, Waltham, MA, US). Quality analysis of the generated sequences was performed using the PHRED platform [59]. The readable sequences were edited using the BIOEDIT 7.0.9 editor (Hall, 1999 - Ibis Biosciences, Carlsbad, CA, US) and compared with reference sequences retrieved from *GenBank* using the BLAST tool (http://www.ncbi.nlm.nih.gov/BLAST/).

Phylogenetic trees were built using software Mega software version 7.0 [60] The generated trees were constructed by the Neighbor-Joining method using the Tamura 3-parameter model, with bootstrap values of 1000 repetitions.

#### Multiple loci VNTR analysis

Multiple locus variable-number tandem repeat analysis (MLVA) was performed according to Salaün et al. [61] using three discriminatory markers usually presented by *Leptospira interrogans* (VNTR loci 4, 7 and 10). The MLVA was performed in all isolates and clinical samples that retrieved no isolation but presented PCR results.

### Ethical statement

The inclusion of all animals in the study was carried out only after owner’s consent. This project was approved by the Research Ethics Committee of the University Santo Amaro - (authorization no. 03/2018). All dogs were treated initially with ampicillin (22 mg/Kg; intravenously every 8 h), or three doses of penicillin G (40,000 U/kg; intramuscularly every 48 h or 72 h) in a hospital environment.

## Data Availability

All data generated or analysed during this study are included in this published article.

## Abbreviations

EDTA: Ethylenediamine tetraacetic acid
EMJH: Ellinghausen McCullough Johnson Harris
MAT: Microscopic agglutination test
MLST: Multilocus Sequence Typing
MVLA: Multiple locus variable-number tandem repeat analysis
PCR: Polymerase Chain Reaction
VNTR: Variable number tandem repeat

## Declarations

All methods were carried out in accordance with relevant guidelines and regulations.
